# Involving self‐help groups in health‐care institutions: the patients’ contribution to and their view of ‘self‐help friendliness’ as an approach to implement quality criteria of sustainable co‐operation

**DOI:** 10.1111/hex.12455

**Published:** 2016-03-28

**Authors:** Stefan Nickel, Alf Trojan, Christopher Kofahl

**Affiliations:** ^1^ Department of Medical SociologyUniversity Medical Center Hamburg‐EppendorfGermany

**Keywords:** co‐operation, participation in health‐care institutions, patient involvement, quality management, self‐help friendliness, self‐help groups

## Abstract

**Background:**

The importance of patient participation and involvement is now widely acknowledged; in the past, few systematic health‐care institution policies existed to establish sustainable co‐operation. In 2004, in Germany, the initiative ‘Self‐Help Friendliness (SHF) and Patient‐Centeredness in Health Care’ was launched to establish and implement quality criteria related to collaboration with patient groups.

**Objectives:**

The objective of this study was to describe (i) how patients were involved in the development of SHF by summarizing a number of studies and (ii) a new survey on the importance and feasibility of SHF.

**Setting and participants:**

In a series of participative studies, SHF was shaped, tested and implemented in 40 health‐care institutions in Germany. Representatives from 157 self‐help groups (SHGs), 50 self‐help organizations and 17 self‐help clearing houses were actively involved. The second objective was reached through a survey of 74 of the 115 member associations of the biggest self‐help umbrella organization at federal level (response rate: 64 %).

**Results:**

Patient involvement included the following: identification of the needs and wishes of SHGs regarding co‐operation, their involvement in the definition of quality criteria of co‐operation, having a crucial role during the implementation of SHF and accrediting health‐care institutions as self‐help friendly. The ten criteria in total were positively valued and perceived as moderately practicable.

**Conclusions:**

Through the intensive involvement of self‐help representatives, it was feasible to develop SHF as a systematic approach to closer collaboration of professionals and SHGs. Some challenges have to be taken into account involving patients and the limitations of our empirical study.

## Introduction

More than three decades ago, Hatch & Kickbusch stated that ‘potential benefits from self‐help are not confined to participants’, but may lie in ‘improvements in the quality and the structure of institutional services and in the behavior of professionals’ (p.195).^1^ Hence, one of the recommendations to the WHO urged national governments ‘to ensure representation of self‐help groups and organizations […] at all relevant levels in decision‐making bodies as one way of ensuring consumer participation in the health care system’ (p.191).^1^ Similar recommendations came from the USA: ‘egalitarian respectful relationships, not superordinate‐subordinate relationships, among self‐help groups and the formal system should be developed’ (p.325).^2^

Since then, many efforts have been made to generally acknowledge self‐help groups (SHG) or ‘voluntary and self‐help organizations’ as mentioned in the WHO's strategy ‘Health 2020’.^3^, ^4^, ^5^, ^6^, ^7^, ^8^ Despite many pleas to extend the role of self‐help, the debate continues on the potentials and problems of SHGs and their collaboration with health‐care professionals and researchers.^5^, ^9^, ^10^

A nationwide self‐help‐support‐system, inclu‐ding around 300 clearing houses for self‐help support, was developed and established over the last 30 years in Germany, predominantly to promote regional SHGs, but also self‐help organizations.^11^^,^ 12 Furthermore, since 2004 at the national level, public and patient involvement in the health‐care system has been legally mandated and to a large degree successfully implemented.^13^^,^ 14

At the meso‐level of single health‐care institutions, however, general provisions rarely exist for the participation and systematic involvement of patients in ‘their’ services; the few measures described in the literature usually deal with provisions for the financial funding of self‐help support.^15^ While *individual* patient participation – specifically in the context of shared decision making – is not uncommon, there is a lack of systematic approaches at the *collective* level, that is, the involvement of patient or self‐help *groups* in health‐care organizations.^16^ The main reason for this can be seen in the observation that Germany's health‐care system is fragmented and divided into different sections, similarly to other health insurance determined ‘Bismarckian’ systems.^17^ With respect to fragmentation, however, the German system is slightly worse because of its federal structure, which additionally increases the complexity and diversity of health‐care structures. This situation is completely different to that in the centralized and tax‐based ‘Beveridge’ systems, for example, in Spain, and particularly in the original, the NHS of the UK, that is probably the most significant prototype.^18^ In the UK, we can find elaborate public and patient involvement strategies and approaches, from the national political level down to local general practice ‘in one pour’ (without judging here to which extent they work or do not work). The NHS strategy ‘The NHS belongs to us all – transforming participation in health and care’ can be mentioned here as a vivid example.^19^ Such a top‐down approach would not be feasible in Germany; therefore, self‐help friendliness (SHF) was developed as an approach to implement wider co‐operation between self‐help associations and health‐care services.^20^^,^ 21 The main goal of SHF is to involve patients as much as possible and is derived from the established slogan of activists in the organizations of disabled and handicapped people: ‘Nothing about me without me’.^22^ Other motives are based on more instrumental purposes to improve patient‐centred health care: (i) to avoid an over‐reliance on the perspective of health professionals and (ii) to include patients in the quality management of health‐care institutions.^23^^,^ 24

The notion of SHF implies a partnership approach (as opposed to the above‐mentioned ‘superordinate–subordinate relationships’) for sustainable collaboration with patients. Our understanding of partnerships is close to Baggott's working definition as ‘a range of collaborative working arrangements, institutions and processes, involving organizations and individuals, that seek to improve the health and wellbeing’ (p.11).^25^ In Germany, the most common umbrella terms for patient associations are ‘self‐help *groups*’ (predominantly for smaller informal groups at local level) and ‘self‐help *organizations’* for the more formally organized non‐profit associations at national or federal state level. Particularly, the latter ones include what in other contexts or countries is called patient advocacy, consumer or user associations, or ‘HCPO’ (Health Consumer and Patient Organizations).

In the previous studies, we have reported on the approach in general,^26^ its potential for more patient‐centredness in outpatient care^27^ and its possible role in a sustainable reorientation of health services, predominantly in health promoting hospitals.^28^ In this study, we focus on the following:

1. the challenges and possibilities of involving patients at the collective level in the SHF approach, that is, representatives of self‐help groups and organizations, by summarizing a number of pilot studies and projects.
2. a recent major study, specifically dedicated to the assessment of the importance and feasibility of quality criteria of good collaboration.

The advantages and shortcomings of both the involvement of self‐help representatives (SHRs) and the empirical study on collaboration criteria will be discussed at the end.

## Challenges and possibilities of involving patients

### Methodical approach: developing SHF as a complex participative action research programme

Our account of the involvement of self‐help associations looks at a series of empirical studies and practice‐oriented development projects with regard to SHF between 2004 and 2013 (see Table 1).^21^, ^29^ The methods of these studies varied widely; the empirical research combined qualitative (expert interviews and focus groups) with quantitative surveys. Whenever possible, patient representatives interacted with health‐care professionals and researchers. Because of the participative approach, the project development and research proceeded stepwise; every incremental step advanced the clarification of the concept and its further implementation. The core group in this process consisted of professional self‐help supporters, social scientists and staff from both health‐care insurance companies and health‐care providers. One milestone was the foundation of a network on SHF in 2009.^30^ The network members had previously been involved in dealing with SHGs in various contexts and functions. They saw their mission as developing and implementing a more systematic approach in health‐care institutions. Health‐care insurance companies played a major role because of their function as funders of pilot projects and applied research. The network's general policies and activities were, and still are, discussed and decided by a steering group of health‐care insurance representatives and a professional self‐help supporter from a ‘federal coordination office’ (www.selbsthilfefreundlichkeit.de).

**Table 1:** Major studies and steps in the development of SHF

Study area (year)	Type of study	Sample	Main results
Hospital, part 1 (2004/2005)	Explorative survey	30 SHO, 20 SH clearinghouses	Participative development of criteria
Hospital, part 2 (2004–2006)	Model project, implementation study	Two hospitals in Hamburg	Testing and final formulation of eight criteria; two hospitals awarded ‘quality seal’
Hospital, part 3 (2008–2010)	Model project, implementation study	31 hospitals in NRW, 17 finishing the process	Process pattern and guidelines for becoming self‐help friendly; 17 hospitals awarded distinction
Public health service (2009–2011)	Delphi method, interactive identification and approval of quality criteria	16 public health departments	10 quality criteria approved by workshop of public health doctors at their annual conference 2011
Ambulatory care (2009–2011)	Model project, implementation study	Nine practices, individual MDs from 8 specialties	Six criteria approved and introduced into quality management manual for doctors in NRW
Rehabilitation (2010–2013)	Model project, implementation study prepared by focus group of 14 SHR	Two rehabilitation hospitals	Five criteria successfully tested; two hospitals awarded distinction; introduction in one national accreditation system planned

### Results: Self‐help representatives in the process of development and implementation of self‐help friendliness

#### SHF in hospital care

The concept of SHF stems from two sources. The first is a former survey of 345 contact persons of 658 SHGs in Hamburg (response rate: 52.4%).^31^ This research showed that most SHGs aimed to change the attitudes of health‐care professionals; nearly half desired a change in institutions. These data pointed to the need for a more systematic approach to implement collaboration.

The second source is the annual meetings of SHG members and medical doctors, jointly organized by the Medical Chamber of Hamburg and the local clearing house for SHGs. In one of these meetings, the notion of ‘self‐help friendly hospitals’ arose; this term was used in an intensive discussion between representatives of Hamburgian hospitals and the clearing house about a formal co‐operation statement between hospitals and SHGs in 2003, and to initiate a model project on SHF in hospitals. In autumn 2004, funds for an explorative research project were granted from the Federal Association of Company Health Insurance Funds (BKK BV).

In 2005, representatives of 30 self‐help organizations with experience of co‐operation, and 20 self‐help clearing houses, answered a questionnaire concerning their wishes, expectations and assessments of different quality criteria and recommendations. Their assessments and recommendations provided a significant basis for the identification of relevant quality criteria for SHF.^32^

The steering group of the model project consisted of the project manager (an experienced former self‐help supporter and consultant for change management), three hospital quality assurance managers, two staff members of the local clearing house and four representatives of SHGs. Scientists of the Hamburg Department of Medical Sociology worked as consultants and moderators. Eight criteria for good colla‐boration between hospitals and SHGs were developed and agreed on:

1. The hospital offers rooms, infrastructure and possibilities for public relations.
2. Patients of the hospital are personally informed about self‐help on a regular basis.
3. The hospital supports public relations of the SHG.
4. The hospital appoints a staff member as a contact person for self‐help.
5. Staff and SHG members meet regularly for information exchange.
6. SHGs are involved in further education/training of staff.
7. SHGs are involved in quality circles and ethics committees.
8. The collaboration is formally agreed on, and the activities will be documented.

A quality circle as mentioned in #7 is a work group of employees or persons involved in a process who meet regularly to investigate and discuss their quality problems to develop solutions and actions for improvement. Most of the criteria refer to improving support for SHGs, while criteria 5–7 aim at permanent involvement of SHGs to improve service quality.

In Hamburg, the implementation of the self‐help criteria was successfully tested in two hospitals. After a formal external audit, the two hospitals were awarded a ‘Quality Seal for Self‐Help Friendliness’ in mid‐2006. Eight members of SHGs had been trained specifically for this task and participated in the on‐site visitation. These quality criteria were published as a brochure with guidelines on how to plan and to implement them.^33^

After the test phase in Hamburg, it took some time until the welfare organization ‘Der PARITÄTISCHE North Rhine‐Westphalia’ became the project holder of the next development project (2008–2010) and founded its own agency for SHF, with a social worker experienced in self‐help affairs. This project produced the following process pattern for becoming self‐help friendly:

1. The agency for SHF (or a self‐help clearing house) contacts and informs the hospitals.
2. First consultation of the agency takes place in the hospital.
3. The agency contracts the hospital and mediates contacts with self‐help clearing houses.
4. The staff of the self‐help clearing houses counsels hospital staff and mediates SHGs.
5. The hospital and SHGs collaborate in a quality circle.
6. Measures to fulfil the quality criteria are put into practice and are part of the internal quality management system.
7. The hospital applies for a certificate (optional).
8. The quality report of the hospital is signed by representatives of SHGs.
9. Certification is awarded (formally documented distinction ‘Self‐Help Friendliness’ in form of a certificate).

The agency conducted consultations with 31 hospitals in North Rhine‐Westphalia (NRW), 17 of which finished the process with a distinction. The requests for consultation overstrained the capacities of the agency (a half‐time social worker).^34^

Both projects in Hamburg and in NRW produced valuable insights and downloadable material that can be used by other hospitals as a blueprint for becoming self‐help friendly (www.selbsthilfefreundlichkeit.de). However, they also showed that some aspects were either too demanding, or even resulted in barriers to further spreading the concept; for example, the agency concept was too expensive. Hence, the present approach is to give a small amount of funding to the existing clearing houses whenever they have an additional workload caused by their support for institutions adopting SHF. Also, the concept of a formal quality seal after an external audit was dropped as it was too time‐consuming, not only for the hospitals but also for the SHRs. Today, health‐care institutions can apply for a distinction of the network ‘Self‐Help Friendliness and Patient Centeredness’ if they are able to present a ‘quality report’ in which patient representatives have certified that at least one measure for each quality criterion has been put into practice and included in the internal quality management system. This has proved most feasible and, at the same time, guarantees that no advertising of SHF claims can be made of any institution without the consent of SHRs.

#### SHF in other areas of the health services

Having been successfully implemented in the hospital area, implementation of the programme was then started in the other health‐care sectors: public health, practices and rehabilitation services.^27^

The *public health sector* was a special case. The process in this area did not fit into the general pattern because the ten quality criteria were an interactive process with professionals from 16 public health departments of local health authorities. Unfortunately, we do not have exact data on the degree of local SHG involvement; therefore, we cannot report here in more detail.^20^

Based on the experience in hospitals, the next project started to develop equivalent criteria for *ambulatory care*. The ten existing recommendations for co‐operation^35^ and the eight quality criteria for inpatient care provided the starting point for formulating criteria for outpatient care (in GPs’ and specialists’ offices) in a concerted action of all relevant players. They produced a consensus document with six criteria that was approved by the Association of Statutory Health Insurance Physicians Westphalia‐Lippe. These criteria are quite similar in essence to those in the hospital sector.

Nine practices (local doctors with their staff) participated in the process; they comprised general practice, gynaecology and obstetrics, internal medicine, urology, ophthalmology, orthopaedics, ENT medicine and paediatrics. The medical and lay persons met four times as a quality circle and jointly developed measures to put the quality criteria into practice. At the end of the process, the success of implementing the criteria was put on record in a quality report which was signed by both parties. The practices were awarded a distinction as being particularly self‐help friendly.^36^ New endeavours are underway to engage networks of doctors, instead of single practices, as partners in further development.

The process in two pilot hospitals for rehabilitation was as participative as in the hospital sector and finished in 2013. The process started with a working group on quality improvement of an umbrella organization of rehabilitation institutions. The resulting preliminary set of criteria was discussed with 14 SHRs in a special workshop for this purpose in December 2011. They produced five quality criteria that were tested and finally confirmed in a model project with two rehabilitation hospitals. The participating SHGs in the model project were as follows: the Interest Group of Contergan Victims, the Federal Osteoporosis Association, the German Multiple Sclerosis Society and a local SHG of stroke patients. At the end, the successful implementation of self‐help friendliness was confirmed by the SHRs and led to a distinction for the two hospitals.

The national network of institutions interested in becoming (or remaining) self‐help friendly has grown steadily since its start in 2009. In November 2015, the network (besides its acting core group, mentioned earlier) had 105 *active members*, among them 39 local self‐help clearing houses, seven self‐help organizations, 27 hospitals and 15 rehabilitation hospitals. Eighteen hospitals and four rehabilitation hospitals currently hold distinctions as self‐help friendly health‐care institutions. In each case, the list contains an enumeration of the collaborating SHGs (about 9, on average).^37^ Forster and Gabe^38^ reported that about 40 hospitals in Austria had taken up the concept by 2011. These figures are encouraging, considering that active dissemination is still at its early stages.

## Exploring the view of self‐help representatives: a survey study

What do SHRs think of the importance and feasibility of the SHF approach as a whole? As we had only qualitative knowledge concerning this issue, we started a quantitative survey in 2012.

### Method

The survey instrument was based on previous research on the evaluation of SHF in hospitals.^39^ Three 4‐item scales related to fostering self‐help capabilities at the *individual* level (informing, involving and empowering patients), together with items published in.^28^ The fourth scale referred to the *collective* level of self‐help friendliness and was called ‘involving SHGs’. This scale consists of ten statements describing the health‐care institution's quality of collaboration with the organized self‐help.^28^ Self‐help representatives were asked to assess the importance and feasibility of the criteria.

Besides a few questions on demographics and the patient's self‐rated severity of own illness (because of confidentiality, there was no question regarding the respondent's or the group's disease), and the tasks and functions of the organization (seven items), the questionnaire included one open‐ended question on the integration of SHF into quality management systems. All items measuring the importance and feasibility of SHF criteria had the same answer scale from 1 to 6 (‘very unimportant’ to ‘very important’ and ‘hardly feasible’ to ‘very feasible’, respectively). Additionally, users could tick the box ‘cannot assess’.

Procedure and respondents: We sent a postal questionnaire to 115 member organizations of the Federal Working Group for Self‐help (BAG SELBSTHILFE), a large umbrella organization of national‐level self‐help organizations for disabled and chronically ill persons. The member organizations could determine themselves who answered the questions. Questionnaires could be sent back either by mail, fax or e‐mail to the research institute; a reminder was posted 2–3 weeks after the first contact. We got 74 responses (64%).

Fifty‐eight percentage of the participating SHRs were male, and 50% were 60 years or older. Only 32 % of the respondents felt free of acute health problems at this point. Most of the SHRs (69 %) were primarily working in functions *within* the organization (e.g. in management or the executive board, or as group leaders), 23 % described their function as inside *and* outside the organization and 8 % understood themselves as patient representatives in other contexts (e.g. political participation and representation).

Descriptive analyses were performed as frequency distributions, cross tables and mean comparisons. Differences between function in the self‐help organization and other characteristics of the respondents (age, sex, education, health status) were statistically tested. The open‐ended question of the survey was analysed by quantitative content analysis.

### Results

#### The importance and feasibility of criteria related to self‐help representatives

The focus of this paper is on the collective level of SHF, that is, on the involvement of patient or SHGs (not only individual patients) in health‐care organizations. Therefore, we will only present our results concerning the subdimension ‘involving SHGs’ (ten items; see Method).

**Figure 1:**
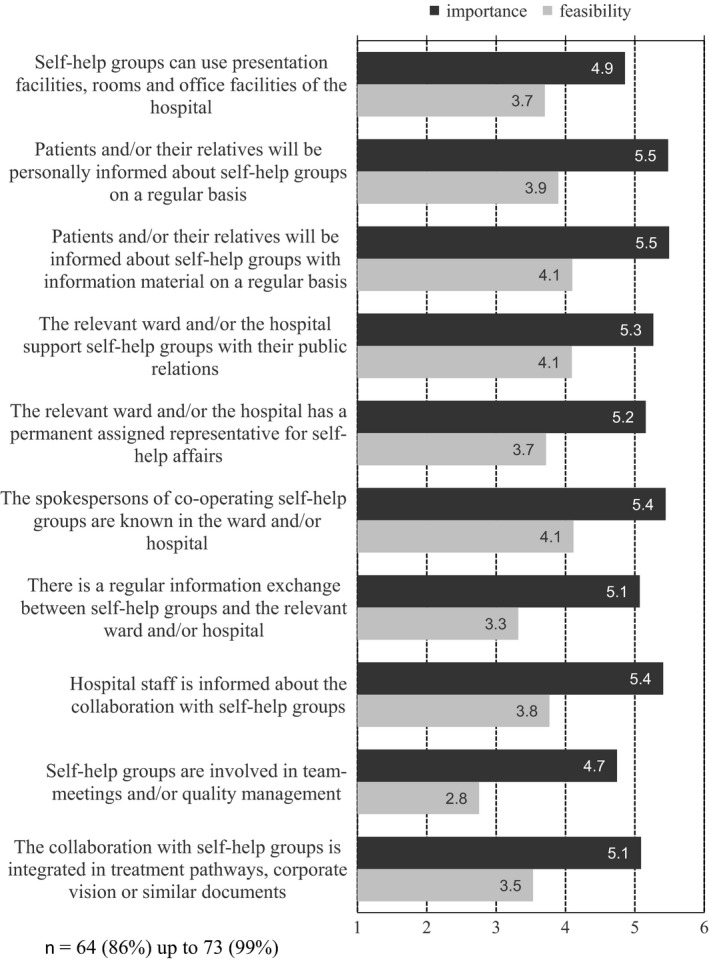
Importance and feasibility of SHF criteria on the collective level (mean: 1 = very unimportant/hardly feasible, 6 = very important/very feasible).

Figure 1 shows the average scores of SHRs for the assessed importance and feasibility of SHF criteria. The *importance* of all quality statements was assessed as very high (means: 4.7 up to 5.5).

The perceived *practical feasibility* of the SHF criteria was rated lower than their importance. Considered to be easy to implement were those types of co‐operation that already exist in many places or can be expected to need little effort, that is, informing patients and/or their relatives about SHGs with relevant material on a regular basis, and supporting SHGs in their public relations (criteria 3 and 4 in Fig. 1). Less easy to implement, however, was the desired involvement of SHGs in team meetings and/or quality management, as well as regular information exchange between SHGs and the relevant ward and/or hospital (criteria 7 and 9).

Additional analyses were carried out to detect possible confounders of the assessments on the importance and feasibility of SHF criteria; we did not find significant differences by sex, age, education or health status of the responders. Only persons with double functions inside and outside their organization made comparatively lower ratings in some points.

#### Integration of self‐help representatives into quality management

**Figure 2:**
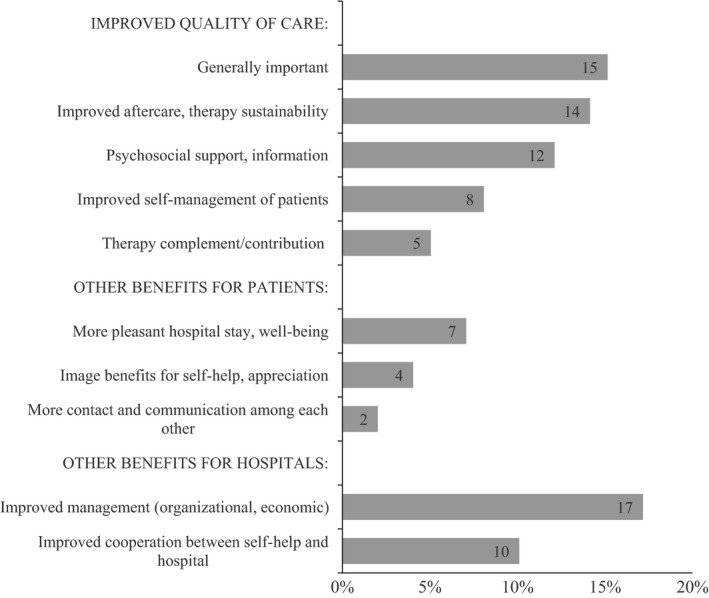
Positive comments on the integration of SHF into the QM system of hospitals (in percent of 99 statements from 58 respondents).

In an open‐ended question, the SHRs were asked: ‘What is your opinion about the implementation of SHF criteria in the quality management systems of hospitals and other health services?’ Negative opinions were very rare, and no clear picture emerged about their focus. Most comments were positive. We have categorized them as shown in Fig. 2. Fifty‐four of 99 comments (55 %) referred to the ‘improved quality of care’. Other benefits for patients are mentioned in 13 comments (13 %). Benefits for hospitals were mentioned 27 times (27 %).

## Discussion and conclusion

### Involving self‐help representatives

Self‐help friendliness claims to be an approach that, with possible variations for other countries, is viable for all health‐care institutions trying to implement on‐going collaboration with patient groups. It is a professional method of sustainably involving patient groups into the running of hospitals and other health‐care institutions. The focus of this paper is on the involvement of the potential beneficiaries of the SHF approach in its development. The merits and problems of the approach in general have been discussed elsewhere.^26^, ^27^, ^28^ As Sarah Hawley has written in her recent HEX editorial: the process matters.^40^ Therefore, we aimed to give an insight into the complexity of patient involvement in the framework of institutional rules and regulations. The development of the SHF approach was different compared to those participative research projects which relate to a specific health topic, such as cancer, or are a limited duration,^41^ a single‐purpose participation (e.g. guideline development^14^) or other clinical case studies.^42^ It is an example of the permanent involvement of representatives of SHGs in a reform programme to give patients a voice. According to the classifications of Nilsen *et al*. in their Cochrane review^43^, the SHF approach can be best characterized as a collaborative process that involves established consumer organizations in different sites and repeated events. Referring to a multidimensional framework stemming from a comprehensive review of public involvement in health research, in our programme the ‘researchers’ degree of engagement’ was high (demonstrated by, e.g., inviting lay groups to participate) for a medium level of public engagement (‘collaboration, consultation’; not ‘control’) (p.76).^44^ In fact, there was a great number of participating actors: until July, 2015, the website of the national network counted 157 regional SHGs, 50 self‐help organizations and 17 regional self‐help clearing houses that had at some point participated in the development and implementation of SHF in the various sectors of health care.^39^

The figures reported earlier show an impressive degree of participation in the development and implementation process.^45^ The interest of self‐help organizations in becoming members of the network is, however, lower (only six of 96 members). Obvious reasons for this are the lack of clearly defined roles and rights within the network, or that they feel represented by the 34 self‐help clearing houses in the network. Self‐help representatives were invited to take a number of roles in the research and development process. Brett *et al*.^46^ recently have provided a summary of the beneficial impacts of patient and public involvement on health‐care research. These impacts were classified in terms of the stages of a comprehensive research programme and will be used to summarize the roles played by SHRs in the development of SHF:

1. *Initial stages of research –* SHRs responded in qualitative pilot studies and helped to identify relevant criteria of good collaboration and to prioritise them; in the initial working group (and later in quality circles), they were equal partners in the formulation of quality criteria and collaborated to develop a kind of ‘quality seal’ (later more simply called ‘distinction’).
2. *Undertaking (applied) research –* Self‐help representatives in pilot studies helped to assess the appropriateness and wording of research instruments; self‐help clearing houses and some self‐help umbrella organizations assisted with recruitment to studies and thus improved response rates; SHRs were responders in interviews and surveys.
3. *Analysis and write‐up –* some SHRs gave feedback regarding preliminary versions to improve understanding and comprehensiveness, particularly in cases of open‐ended questions.
4. *Dissemination and implementation* – there was little help with the dissemination, but a major impact on the implementation of SHF: SHRs were auditors in the accreditation process for the first self‐help friendly hospitals, advisors and/or participants in workshops and quality circles; and, most important for sustainability, they were and are witnesses and controllers for the granting and renewal of distinctions.

Other than in the review quoted, we registered the most important beneficial impacts in the last phases ‘implementation’ and ‘maintenance’. Nevertheless, ‘challenging impacts’ found in the review^46^ (p. 644) could be observed in our research programme:

1. Difficulties in recruiting a diverse range and representative sample of SHRs to a project.
2. Sessions being overshadowed by personal experience stories, when the aim was to discuss research topics.
3. Increased time and cost owing to the practical aspects of planning and discussing with the SHRs.
4. Training and education for both SHRs and staff from self‐help clearing houses.

Whereas these points can be regarded as challenges that are compensated for by the benefits of participation, we also have to mention some more severe limitations:

1. Except for the first stimulus from SHGs in the annual meetings organized by the Medical Chamber of Hamburg, the whole process was initiated and driven by researchers and funders.
2. Participation was not feasible in all steps of the programme.
3. Although we tried to involve a broad range of SHRs, we had to accept that only a limited number of SHRs were prepared to participate.
4. Roles were restricted to particular modes of contributing to the next step in the programme; there was no continuity in the representation of self‐help.

These points imply that participation was incomplete in many respects. This problem is not unique, and some researchers conclude that a fully participatory project may be unrealistic.^46^

Two other aspects are unsatisfactory: firstly, there are general concerns about the representativeness and legitimacy of SHGs and – at higher organizational levels – about their independence, particularly from the drug industry.^6^ Additionally, minorities such as marginalized patients or patients with rare diseases possibly may not obtain a sufficiently loud voice in the whole process.

The second aspect is related to the poor evidence for measurable impacts of patient and public participation,^46^, ^47^ for a variety of reasons, for example, lack of continuity and identifying only weak influence.^48^ In a qualitative study on the implementation process of SHF in Austria, positive effects were mainly seen in connection with the better quality of co‐operation with patients, better visibility and acknowledgement of the SHGs, and increasing patient‐centredness in hospitals. The interviewed experts did not see any disadvantageous effects.^38^, ^49^

Another challenging issue is the transferability of the approach to other countries. At least in terms of the mere quantity and nationwide coverage, there is probably no other country in the world providing such extensive professional support for patients and SHGs at all levels than Germany. These self‐help specific characteristics of the German health‐care system have certainly facilitated the present acknowledgement and appreciation of SHF. Transferability seems to be no problem in a system similar to that in Germany, such as in Austria, where about 40 hospitals have gone through the process to become self‐help friendly.^39^ In other countries with other systems, new creative ways have to be explored for the development of fruitful partnerships between patients and professionals. Further research on the issue of transferability is needed.

### The views of self‐help representatives on the importance and feasibility of SHF criteria

Second objective of this paper was our recent major survey of SHRs’ views. The high values for importance were expected, specifically because the dimensions and criteria were formulated in accordance with them. The values concerning practical feasibility can be regarded as cautious optimism, as they mostly lie in the upper half of the scale. Only two values are below the middle of the 6‐point scale (2.8 and 3.3), namely involvement in meetings and/or quality management, and regular information exchange. This indicates that SHRs are rather sceptical regarding to what degree ‘true’ participation might be possible. These two criteria certainly deserve particular attention in any future development.

Although there was a wide variety of representatives from nationwide self‐help organizations included in the study, the sample cannot be regarded as being representative, neither for the whole landscape of the self‐help sector in Germany (especially the up to 100 000 SHGs) nor the professional health service institutions and the involved staff. Unfortunately, we have no information on the diseases of the respondents. However, the surveyed member organizations of the BAG SELBSTHILFE cover a wide range of illnesses, disabilities and mental disorders. Although not completely comparable, we would like to mention that a similar survey with quality managers in hospitals about the criteria's importance and practicality showed similar results.^50^ Additionally, the participating SHRs were not typical, insofar as they might have been more than usually motivated to collaborate with health‐care services.

Another shortcoming is the restriction of the study to the self‐help and patient organizations’ points of view on *collaboration* as a major domain of patient‐centredness in health services. Consequently, other important aspects for the well‐being of patients might remain untackled (e.g. communication, patient‐reported outcomes).^51^

### Concluding remarks

Collaboration with self‐help associations is voluntary; therefore, the preparedness of health‐care institutions is a necessary prerequisite for better integration of patient and consumer organizations into the daily routines of health services. However, whereas ‘normal’ institutions demand the adaption of patients to the institution, self‐help friendly institutions are prepared to act the other way round, allowing participation and adapting to patients’ wishes and needs. Hypothetically, collaboration could lead not only to (legitimate) opposing points of view and new challenges, but might also provide a podium for ‘gadflies’ and destructive critics. During the course of our research, however, we did not hear any complaints of this kind from the participating institutions. The most plausible reason seems to be that the dependency of patients on ‘their’ professional institutions is so high that open conflict generally will be avoided.

‘Self‐help friendliness’ is a catchy label, quite suitable as a short title and attractive for the public relations of health‐care institutions. Nevertheless, taking a second and more intensive look at the concept, it has a somewhat paternalistic connotation (‘health‐care institutions grant friendliness’). We should emphasize that SHF is intended as an example of Rabeharisoa's idea of a ‘partnership model’, which was meant to describe collaboration with patient organizations as equal partners in scientific research.^52^ Particularly in case of rare diseases, this model may lead the way for a new era by bridging the gap between public research, which is at risk to overlook patients’ demands and expectations, and market‐driven research, which confines research projects to those profitable enough to justify private investments.^53^

The development of SHF was certainly value‐driven. Gradinger *et al*. provided an impressive account of the various values attached to public involvement in research (p.7).^54^ Three clusters emerged: ‘normative’, ‘substantive’ and ‘process value system’, each with five subdimensions. Following their recommendation to ‘make explicit one's own values‐based rationale for public involvement’ (p.11), we would highlight those of empowerment (from the normative system), increasing quality, appropriateness and credibility (substantive value system), and partnership and respect (process value system).

In spite of these genuinely positive values, one should keep in mind Baggott's warning of taking a too ‘rose‐tinted’ view of partnerships (p.169): partnerships working with the generally less powerful self‐help organizations can easily be misused to enhance the power of professionals and protect (economic) institutional interests, instead of establishing collaborations of equals at eye level.^25^

## Sources of funding

Most of the studies mentioned in part 3 were financially supported by the Federal Association of Company Health Insurance Funds (BKK Bundesverband), Germany. The development and testing of the instrument for the measurement of self‐help‐oriented patient‐centredness in hospitals (SelP‐K), mentioned in part 3, were conducted within a research project ‘Quality concept ‘Self‐Help Friendly Hospital’ as an approach for patient centred participative care’, funded by the Federal Ministry for Education and Research, 2008/01‐2011/12. The study reported in part 4 was funded by the Bundesarbeitsgemeinschaft SELBSTHILFE (Federal Working Group for Self‐help of People with Disabilities and Chronic Illness; shortly: BAG SELBSTHILFE), the German umbrella organization for self‐help associations on the federal level.

## Conflict of interest

One of the authors (AT) is member of the volunteer Steering Committee of the German Network ‘Self‐Help Friendliness and Patient Centeredness’.
